# Clinical analysis of conversation effectiveness and burden in dyads of native Cantonese speakers with Fronto‐temporal dementia

**DOI:** 10.1002/alz70858_107730

**Published:** 2025-12-26

**Authors:** Lorinda Li‐Ying Kwan Chen, Hoi Lam Kaitlyn NG, Joshua Tsoh, Andrew L.T. Chan, Boon Lead Tee

**Affiliations:** ^1^ The Education University of Hong Kong, Tai Po, New Territories, Hong Kong; ^2^ Prince Of Wales Hospital and Shatin Hospital, Shatin, New Territories, Hong Kong; ^3^ Department of Special Education and Counselling, The Education University of Hong Kong, Hong Kong, Hong Kong; ^4^ The Education University of Hong Kong, Tai Po, new Territories, Hong Kong; ^5^ Prince of Wales Hospital and ShaTin Hospital, Ma On Shan, New Territories, Hong Kong; ^6^ Divisions of Neurology and Geriatrics, Department of Medicine, Queen Elizabeth Hospital, Central Kowloon, Hong Kong; ^7^ Memory and Aging Center, Department of Neurology, University of California San Francisco, San Francisco, CA, USA; ^8^ Memory and Aging Center, Weill Institute for Neurosciences, University of California San Francisco, San Francisco, CA, USA; ^9^ Memory and Aging Center, UCSF Weill Institute for Neurosciences, University of California, San Francisco, San Francisco, CA, USA; ^10^ Global Brain Health Institute, University of California, San Francisco, San Francisco, CA, USA; ^11^ CiJi hospital, HuaLien, Taiwan; ^12^ University of California, San Francisco, San Francisco, CA, USA; ^13^ Buddhist Tzu Chi General Hospital, Hualien, Taiwan

## Abstract

**Background:**

Examining conversation breakdown in individuals with fronto‐temporal dementia (FTD) has proven effective for identifying conversational troubles, their repairs, and the effectiveness of such repairs. This study appraised conversation effectiveness by describing the contributions of Hong Kong Cantonese‐speaking individuals with behavior‐variant fronto‐temporal dementia (bvFTD) and/or primary progressive aphasia (PPA) along with their communication partners. Additionally, it explored the troubles and repair behaviors occurring in dyadic conversations.

**Method:**

Sixteen participants were recruited, comprising 4 individuals with bvFTD and/or PPA and their partners (*N* = 8), alongside 4 control dyads (*N* = 8). Dyadic conversations covering three topics (a picture description, a faux pas scenario, and a problem‐solving discussion of an unknown object) were video recorded and transcribed. Trouble‐indicating behaviors (TIBs) and repair behaviors were classified, while measurements included the quantity of words, turns, and length of turns.

**Result:**

The analysis revealed that, based on the percentage of turns, individuals with bvFTD and/or svPPA participated in conversations equally, whereas those with nfvPPA were less equally engaged. Individuals with bvFTD showed decreased theory of mind abilities, resulting in an increased frequency of TIBs and less consistent interactions. Notable distinctions were observed in the conversational patterns of four bvFTD and/or PPA dyads, indicating features like effortful speech in nfvPPA individuals and the absence of nouns in svPPA individuals.

**Conclusion:**

Our findings indicate that Cantonese‐speaking individuals with bvFTD and/or PPA actively engage in situational conversations by effectively identifying and addressing troubles, as well as employing appropriate repair strategies to enhance conversation effectiveness. This challenges the notion that bvFTD or PPA is solely linked to communication difficulties. Rather, individuals with these conditions demonstrate the capability to engage equally in discussions and adjust their communication strategies tactfully. This understanding can significantly inform the development of interventions that enhance communication skills and the quality of life for people with FTD.

